# Recent advances of anti-angiogenic inhibitors targeting VEGF/VEGFR axis

**DOI:** 10.3389/fphar.2023.1307860

**Published:** 2024-01-04

**Authors:** Lei Wang, Wang-Qing Liu, Sylvain Broussy, Bingnan Han, Hongming Fang

**Affiliations:** ^1^ Department of Oncology, Zhejiang Xiaoshan Hospital, Hangzhou, China; ^2^ Zhejiang Provincial Key Laboratory of Silkworm Bioreactor and Biomedicine, College of Life Sciences and Medicine, Zhejiang Sci-Tech University, Hangzhou, China; ^3^ CiTCoM, CNRS, INSERM, Université Paris Cité, Paris, France

**Keywords:** VEGF, VEGFR, angiogenesis, anti-angiogenic, inhibitors

## Abstract

Vascular endothelial growth factors (VEGF), Vascular endothelial growth factor receptors (VEGFR) and their downstream signaling pathways are promising targets in anti-angiogenic therapy. They constitute a crucial system to regulate physiological and pathological angiogenesis. In the last 20 years, many anti-angiogenic drugs have been developed based on VEGF/VEGFR system to treat diverse cancers and retinopathies, and new drugs with improved properties continue to emerge at a fast rate. They consist of different molecular structures and characteristics, which enable them to inhibit the interaction of VEGF/VEGFR, to inhibit the activity of VEGFR tyrosine kinase (TK), or to inhibit VEGFR downstream signaling. In this paper, we reviewed the development of marketed anti-angiogenic drugs involved in the VEGF/VEGFR axis, as well as some important drug candidates in clinical trials. We discuss their mode of action, their clinical benefits, and the current challenges that will need to be addressed by the next-generation of anti-angiogenic drugs. We focus on the molecular structures and characteristics of each drug, including those approved only in China.

## 1 Introduction

Angiogenesis is a complex process including endothelial cell (EC) proliferation, migration, vascular tube formation, tube ligation, and finally formation of the new blood vessels from pre-existing ones (Risau, 1997). Usually, it is a normal physiological circumstance to supply nutrients and oxygen during embryonic development and wound healing (Chung et al., 2010). In the 1970 s, Judah Folkman observed the correlation of angiogenesis and solid tumor growth, which indicated that the growth of tumor beyond a critical size of 1-2 mm^3^ needed formation of new vessels around it (Folkman, 1971). This process is also called sprouting angiogenesis. He proposed to prevent tumor growth by inhibiting angiogenesis, which is nowadays named antiangiogenic therapy (Folkman, 1972; 1975). Later, angiogenesis was also observed to be related to a wide range of other pathological conditions, including arthritis, retinopathies, atherosclerosis, and endometriosis (Carmeliet, 2003; Chung and Han, 2022). From there, the study of the mechanism of angiogenesis and the development of antiangiogenic drugs has been an intense research subject.

Normally, angiogenesis is finely regulated by diverse endogenous pro- and anti-angiogenic factors (Ferrara and Kerbel, 2005; Kopec and Abramczyk, 2022). However, overexpression of pro-angiogenic factors will break this balance and result in pathological angiogenesis (Teleanu et al., 2019). For example, tumor cells can particularly produce pro-angiogenic factors in the nearby microenvironment and trigger new blood vessel construction to supply nutrients required for tumor growth and metastasis (De Palma et al., 2017). In diabetic macular edema (DME) and neovascular age-related macular degeneration (nAMD) conditions, pro-angiogenic factors promote the growth of abnormal vessels in the retina (Regula et al., 2016).

Well-known pro-angiogenic factors include vascular endothelial growth factor (VEGF), basic fibroblast growth factor (bFGF), epidermal growth factor (EGF), platelet-derived growth factor (PDGF), insulin-like growth factor, transforming growth factor (TGF), and angiopoietin (Marech et al., 2016). Among them, the VEGF has been reported as having a crucial role not only in angiogenesis, but also in lymphangiogenesis (Ferrara and Davis-Smyth, 1997). The VEGFs are a family of homodimeric glycoproteins, including VEGF-A, VEGF-B, VEGF-C, VEGF-D and placental growth factor (PlGF) in mammals (Ferrara, 2016). Among them, VEGF-A is the first angiogenic factor that was identified and characterized among the VEGF family (referred to as VEGF in this review). VEGF-A has numerous distinct isoforms, such as VEGF-A_121_, VEGF-A_165_, VEGF-A_189_, and VEGF-A_206_. Among them, the most active one in angiogenesis is VEGF-A_165_. It is critical not only in physiological angiogenic processes, such as in development of embryonic vascularization, in skeletal morphogenesis and growth, but also in pathological angiogenesis including tumor cell metastasis (Ceci et al., 2020). PlGF is the second discovered VEGF-family member, which is dispensable in physiological angiogenesis processes and in pathological angiogenesis (Fischer et al., 2008). VEGF-B was reported to be important in inflammatory angiogenesis (Mould et al., 2003). VEGF-C and VEGF-D are observed to be highly expressed during the embryo development, also playing a role in angiogenesis, but more importantly in lymphangiogenesis (Stacker et al., 2001; Karkkainen et al., 2004; Rauniyar et al., 2018).

**FIGURE 1:**
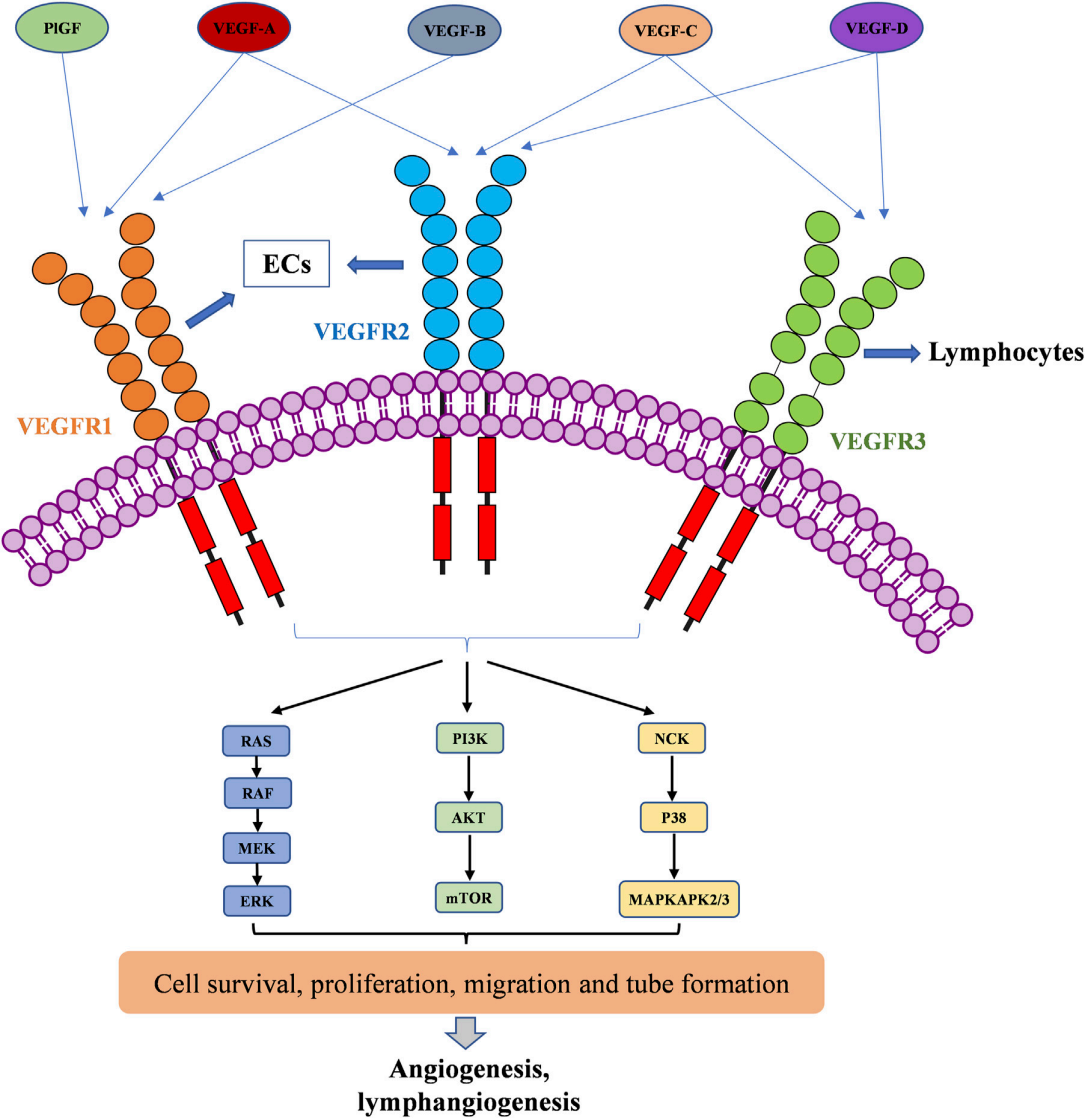
VEGFR activation by the VEGF family of growth factors; and resulting downstream signaling pathways to angiogenesis and lymphangiogenesis.

The VEGF family binds to 3 TK receptors, VEGFR1, VEGFR2, VEGFR3, and two coreceptors, neuropilins 1 and 2 (NRP1 and NRP2) (Ferrara, 2016). The VEGFs and VEGFRs have a crossed binding relationship, which stimulate the activation of different receptors to induce angiogenesis and lymphangiogenesis (Figure 1). VEGF-A binds both VEGFR1 and VEGFR2, VEGF-B and PlGF only bind to VEGFR1, while VEGF-C and VEGF-D bind to VEGFR2 and VEGFR3 (Apte et al., 2019). VEGFR1 and VEGFR2 are mainly expressed on vascular endothelial cells (ECs), but also on some types of cancer cells. The VEGFR2 has been clearly studied as the main VEGF signaling receptor. The binding of VEGF to VEGFR2 stimulates strong downstream signaling to promote EC proliferation, migration and tube formation (Dakowicz et al., 2022). However, the exact mechanism of VEGFR1 signaling is not fully understood. The binding of VEGF to VEGFR1 stimulates much weaker downstream signaling. As a result, it is sometimes considered as a decoy receptor for VEGF (Weddell et al., 2017). On the other hand, VEGFR1 can also activate downstream signaling especially in pathological conditions, such as in several cancers, where VEGFR1 was observed to be overexpressed (Jayson et al., 2016; Weddell et al., 2017). VEGFR3 is specifically expressed on lymphatic ECs (Monaghan et al., 2021). VEGFR3 binds to VEGF-C and VEGF-D to mediate lymphangiogenesis and developmental angiogenesis, and it is considered to be a more important regulator in lymphangiogenesis (Varney and Singh, 2015; Heinolainen et al., 2017). Although VEGFR1 and VEGFR3 seem less important in transporting angiogenic downstream signaling than VEGFR2, when in extreme condition, such as solely inhibiting VEGFR2 signaling pathway, VEGFR1 and VEGFR3 are able to substitute VEGFR2 angiogenic activity (Karaman et al., 2022). Therefore, inhibition of multi-VEGFR signaling was considered to achieve better anti-angiogenic activity (Ceci et al., 2020).

The VEGFRs are dimeric immunoglobulins (Ig) homologues containing seven extracellular domains, a transmembrane domain, and an intracellular tyrosine kinase (TK) domain (Karaman et al., 2018). The VEGF binds to the extracellular domains of VEGFRs, mainly domain 2 and domain 3 (Wiesmann et al., 1997; Iyer et al., 2010; Brozzo et al., 2012). The binding of VEGFs and VEGFRs promotes the dimerization of receptors and is followed by intracellular approaching and activation of TK domains (Ferrara et al., 2003). Then, the downstream signaling transduction through RAF/MEK/ERK, PI3K/AKT/mTOR, and NCK/p38/MAPKAPK2/3 pathways affects endothelial cell migration, proliferation, tube formation and survival (Kroll and Waltenberger, 1997; Wang et al., 2020; Mabeta and Steenkamp, 2022). The activation of RAS/RAF/MEK/ERK signaling pathway will directly promote EC survival and proliferation (Takahashi et al., 1999; Mineur et al., 2007). The PI3K/AKT signaling can activate the production of endothelial nitric oxide synthase (eNOS) and its release in blood vessels to increase vascular permeability (Zachary, 2003), it is responsible for the expression of Cdc42, Rho, and Rac proteins, which are required for tumor cell invasion and metastasis (Jiang and Liu, 2009; Pang et al., 2011; Graupera and Potente, 2013). VEGF can also activate the P38/MAPKAPK2/3 signaling pathway through NCK binding to induce a change in EC cytoskeleton and promote cell migration, thereby resulting in new tube formation (Jiang et al., 2020).

Since the “neovascularization” hypothesis of Folkman in 1971 (Folkman, 1971), antiangiogenic therapy has gained considerable attention and is nowadays a proved therapy to treat different cancers, DME and nAMD. It consists of disrupting the vascular supply by blocking the pro-angiogenic factors or inhibiting activity of their receptors with pharmacological agents (Ramjiawan et al., 2017). Dozens of antiangiogenic drugs have been approved by the FDA including antibodies, fusion proteins and small molecules targeting VEGF/VEGFR axis and its downstream signaling pathway (Qi et al., 2022). Antibodies and fusion proteins have a large molecule size, hence are not able to cross the cell membrane. They either bind to VEGF or VEGFR on the extracellular compartment to inhibit angiogenesis by blocking the interaction between VEGF and VEGFR (Liberski et al., 2022). Conversely, all approved drugs targeting VEGFR TK and its downstream signaling pathway are small molecules (Li et al., 2022). Antibodies and fusion proteins can be used as anti-cancer drugs and drugs to treat DME and nAMD (ElSheikh et al., 2022). However, all small molecules targeting VEGF/VEGFR axis or downstream signaling pathway are anti-cancer drugs, none of them was approved to treat DME and nAMD yet, because of their safety issues (Poor et al., 2022).

**FIGURE 2:**
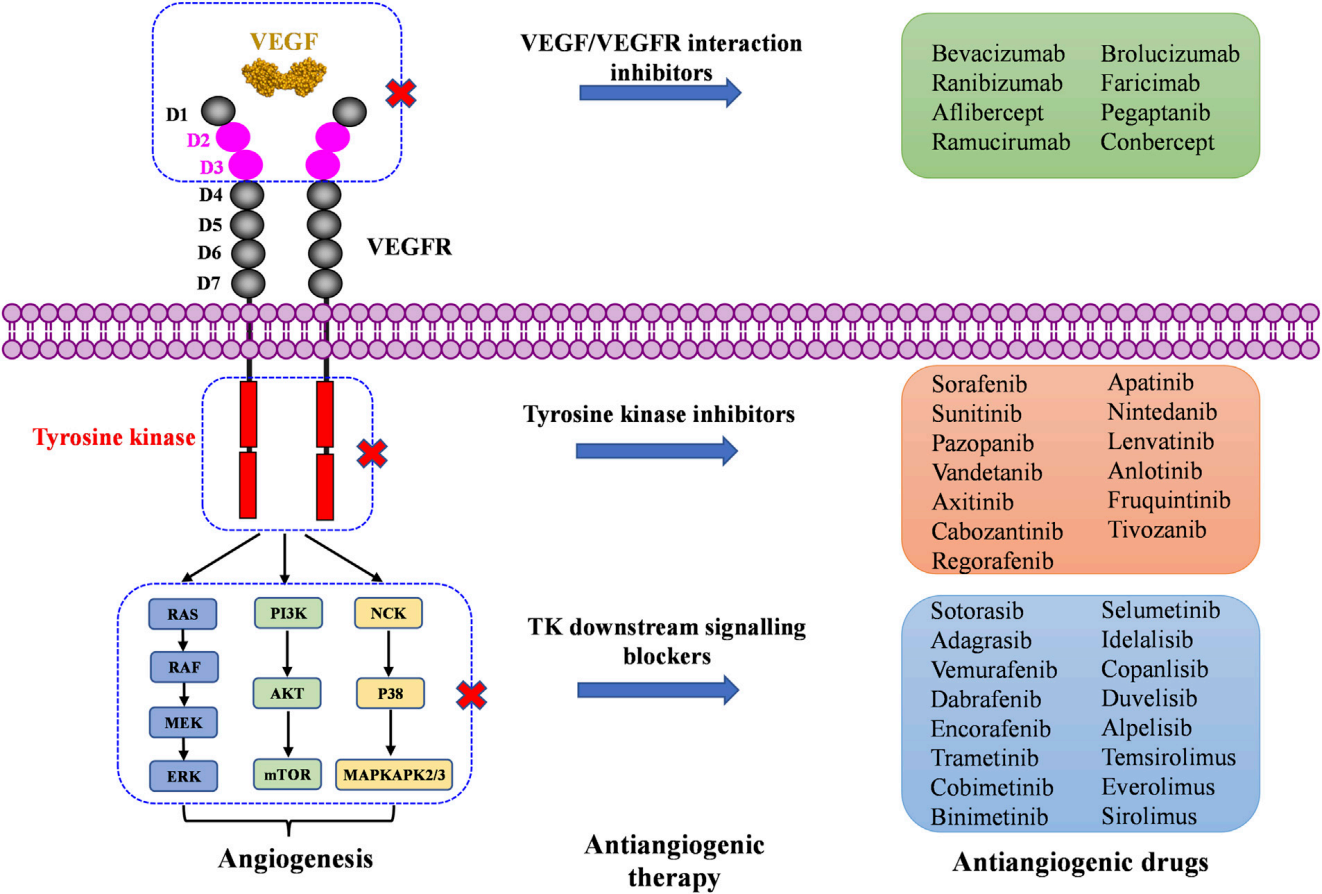
Marketed drugs targeting the VEGF/VEGFR axis and its downstream signaling pathway.

In this review, we present the development of these marketed antiangiogenic drugs involved in the VEGF/VEGFR axis and its downstream signaling pathways (Figure 2), as well as some important drug candidates in clinical trials. We discuss their mode of action, their clinical benefits and adverse effects, and the current challenges that will need to be addressed by the next-generation of anti-angiogenic drugs. We focus on the molecular structures and characteristics of each drug, including those approved only in China. The reader is also referred to some recent reviews on mechanisms of angiogenesis and their applications in oncology and in ophthalmology (ElSheikh et al., 2022; Mabeta and Steenkamp, 2022; Liu et al., 2023; Cao et al., 2023; Ghalehbandi et al., 2023). This overview, while presenting emerging new drugs and targets, may provide insights and perspectives for the future development of antiangiogenic drugs based on VEGF/VEGFR axis and its downstream signaling pathways.

## 2 Inhibitors targeting VEGF/VEGFR interaction

**FIGURE 3:**
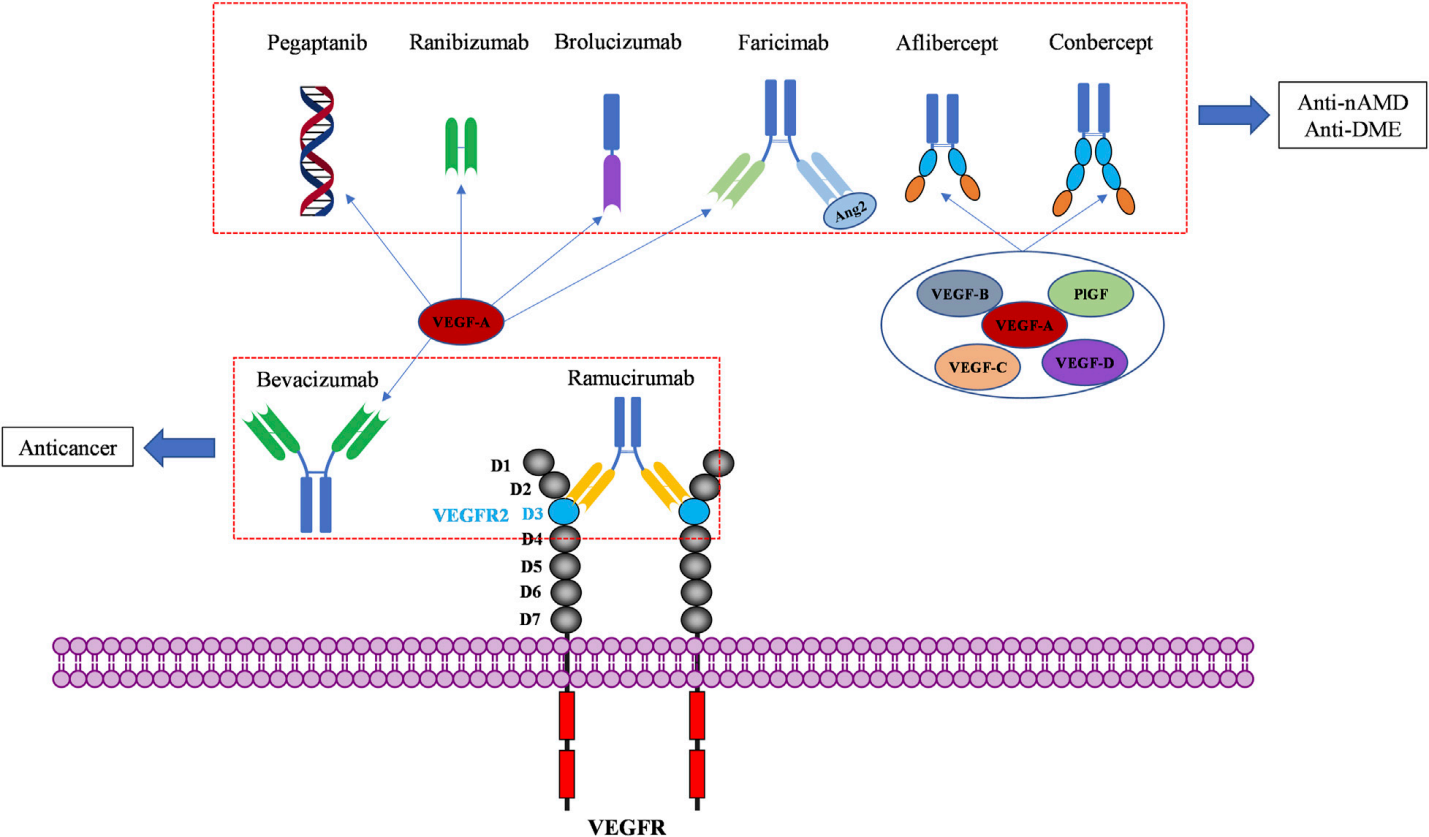
Macromolecular drugs targeting the extracellular compartment of the VEGF/VEGFR axis for anticancer and ocular therapies.

VEGF binds to extracellular domain of VEGFR to activate VEGFR intracellular signaling. Molecules blocking the interaction of VEGF and VEGFR can prevent the activation of VEGFR signaling, resulting in anti-angiogenic affects. To date, seven VEGF/VEGFR interaction inhibitors have been approved by the FDA and one (Conbercept) was approved in China by the National Medical Products Administration (NMPA). Two of these biomolecules are approved as anti-angiogenic drugs to treat cancers, six of them to treat ocular vascular diseases (Figure 3; Table 1). None of them is a small molecule, because the large and flat interaction interface between VEGF and VEGFR is difficult to target.

**TABLE 1:** Approved anti-angiogenic drugs targeting VEGF/VEGFR interactions.

Name	Chemical properties	Targets	First approval	Approved indications
Bevacizumab	Full-length humanized monoclonal antibody	VEGF-A	2004 by FDA	GBM, NSCLC, CRC, BC, RCC, OC
Pegaptanib	Pegylated polynucleotide aptamer	VEGF_165_	2004 by FDA	nAMD
Ranibizumab	Recombinant humanized IgG kappa isotype monoclonal antibody fragment	VEGF-A	2006 by FDA	nAMD, DME, DR
Aflibercept	Recombinant fusion protein consisting of VEGFR-1 D2 and VEGFR-2 D3	VEGF-A/B/C/D, PlGF	2011 by FDA	nAMD, DME, RVO, DR, ROP
Conbercept	Recombinant fusion protein consisting of VEGFR-1 D2 and VEGFR-2 D3-D4	VEGF-A/B/C/D, PlGF	2014 by NMPA	nAMD, DME
Ramucirumab	Humanized monoclonal antibody	VEGFR2	2014 by FDA	CRC, NSCLC, GEJ, HCC
Brolucizumab	Humanized single-chain fragment antibody	VEGF-A	2019 by FDA	nAMD
Faricimab	Bispecific monoclonal antibody	VEGF-A, Ang-2	2021 by FDA	nAMD, DME

### 2.1 Bevacizumab

Bevacizumab was the first anti-angiogenic drug, approved in 2004 in the United States (US) (Muhsin et al., 2004). It is a full-length humanized monoclonal antibody that binds to circulating VEGF, preventing the interaction of VEGF with VEGFR and inhibiting the activation of VEGFR signaling pathways that promote angiogenesis (Ferrara and Adamis, 2016). Early clinical development of bevacizumab was focused on several solid tumor types associated with VEGF driven angiogenesis, including non-small-cell lung cancer (NSCLC) (Seto et al., 2006), metastatic colorectal cancer (CRC) (Des Guetz et al., 2006), metastatic breast cancer (BC) (Foekens et al., 2001), glioblastoma multiforme (GBM) (Flynn et al., 2008), and ovarian cancer (OC) (Choi et al., 2015). These clinical studies have conducted to bevacizumab approval in a wide range of cancer indications (Garcia et al., 2020). In clinical therapy, the most frequently observed adverse events under bevacizumab treatment are hypertension, fatigue, asthenia, diarrhea and abdominal pain. Complications of surgery and wound healing can happen, and rare cases of severe or fatal hemorrhage have been reported (Garcia et al., 2020). These side effects can be usually managed with standard blood pressure monitoring and treatments with antihypertensive drugs. Later, bevacizumab was demonstrated to have better clinical benefits in combination with chemotherapy in cancer treatment (Wahid et al., 2016). Interestingly, bevacizumab was recently approved in combination with atezolizumab, an immunotherapeutic agent (in addition to chemotherapy). The effectiveness of this combination of an anti-angiogenic and an immunotherapy approach can be explained by the angiogenesis-independent role of VEGF in immune modulation (Motz and Coukos, 2011; Ramjiawan et al., 2017; Socinski et al., 2018). Bevacizumab is now the most widely used antiangiogenic drug, marketed in 134 countries worldwide. Due to its mode of action, bevacizumab was also investigated “off-label” in treatment of nAMD and DME (Moshfeghi et al., 2006; Schmid-Kubista et al., 2009; Chakravarthy et al., 2013; Schauwvlieghe et al., 2016). Its cost is lower than the cost of newer anti-VEGF agents, but it has not been officially approved in retinopathies.

### 2.2 Pegaptanib

Pegaptanib is a pegylated polynucleotide aptamer that selectively binds to VEGF-A_165_ (an isoform in which the C-terminal part is present) to inhibit angiogenesis and vessel permeability (Ng et al., 2006; Zhou and Wang, 2006). It was approved in 2004, as the first antiangiogenic agent for the treatment of nAMD (Doggrell, 2005). It is administrated by intravitreal (IVT) injection and mostly caused not serious ocular side effects such as eye pain, corneal edema, blurred vision, and non-ocular side effects such as headache, nausea, and diarrhea (Van der Reis et al., 2011). It was well tolerated and effective in patients with nAMD (Kourlas and Schiller, 2006). However, it is not commonly used in clinical practice today because it targets only one isoform of VEGF, and is replaced by more effective anti-VEGF drugs like ranibizumab and aflibercept (Lytvynchuk et al., 2015; Battaglia Parodi et al., 2018).

### 2.3 Ranibizumab

Ranibizumab is a recombinant humanized IgG kappa isotype monoclonal antibody fragment derived from bevacizumab (Chatziralli, 2021). It contains only the antigen-binding fragment (Fab) of bevacizumab. Despite being much smaller than bevacizumab, it has a higher affinity for VEGF (Chen et al., 1999). It was designed specifically for intravitreal administration (IVT) to treat neovascular ocular diseases due to its smaller size, which enables it to diffuse from the vitreous into the retina and choroid (Ferrara et al., 2006; Campochiaro, 2007). Ranibizumab is considered to be safer than bevacizumab, because of its rapid systemic clearance in the body (Dervenis et al., 2017). It has much shorter half lifetime (2–4 days) than bevacizumab (about 3 weeks). Besides, the absence of a fragment crystallizable portion (Fc) prevents binding complement-associated intraocular inflammation after IVT (Ferro Desideri et al., 2019). Ranibizumab was initially approved by the FDA in 2006 for the treatment of nAMD and DME. The common side effects of ranibizumab reported are mainly not serious, similar to those of pegaptanib, such as eye pain and corneal edema, which were attributed to its administration by intravitreal injection (Lee and Shirley, 2021).

### 2.4 Aflibercept

Aflibercept is a recombinant fusion protein consisting of two fragments: the second extracellular domain (D2) of VEGFR1 and the third extracellular domains (D3) of VEGFR2, both fused to the Fc portion of human immunoglobulin G (Ciombor et al., 2013). It neutralizes multiple VEGFR1 and VEGFR2 ligands, including VEGF-A, VEGF-B, and PlGF to exhibit antiangiogenic effects. It was approved by the FDA in 2011 for the treatment of maculopathy and metastatic CRC (Chung and Pherwani, 2013). In cancer treatment, Chiron et al. showed that aflibercept had higher tumor suppressor activity than bevacizumab in patient-derived xenograft (PDX) models of colorectal cancer (Chiron et al., 2014). As an ophthalmic agent, aflibercept was indicated for the treatment of nAMD, DME, macular edema following retinal vein occlusion (RVO), diabetic retinopathy (DR), and retinopathy of prematurity (ROP). Its mode of action has been confirmed as the inhibition of choroidal neovascularization induced by overexpressed VEGFs (Anguita et al., 2021).

### 2.5 Conbercept

Conbercept is also a recombinant anti-VEGF protein comprising the extracellular domains of VEGFR1 and VEGFR2, fused to the Fc of human IgG. It is engineered from a full human cDNA sequence in Chinese hamster ovary cells (de Oliveira Dias et al., 2016). The difference between conbercept and aflibercept is that conbercept consists of the third and the fourth extracellular domain of VEGFR2, while aflibercept incorporates only the third extracellular domain of VEGFR2 (de Oliveira Dias et al., 2016). The fourth extracellular domain of VEGFR2 does not directly participate in binding with VEGF, but it facilitates receptor dimerization and improves the association of VEGF. As a result, conbercept was reported to have an affinity 50-fold higher than bevacizumab on binding to VEGF (Cai et al., 2018). However, until now, conbercept is only approved in China (since 2014) by the NMPA of China for the treatment of nAMD and DME. No intraocular inflammation, retinal or vitreous hemorrhage, or systemic complication have been reported (Qi et al., 2019). It has not yet been granted approval from the FDA or the European Medicines Agency (EMA) yet.

### 2.6 Ramucirumab

Ramucirumab is a humanized monoclonal antibody (IgG) targeting the extracellular domain of VEGFR2, approved in 2014. It is now used for the treatment of advanced or metastatic gastric, or gastro-esophageal junction (GEJ) adenocarcinoma, hepatocellular carcinoma (HCC), CRC, and NSCLC (Vennepureddy et al., 2017; Khan and Shah, 2019; Syed, 2020). Ramucirumab binds specifically to VEGFR2, more precisely on the domain 3 of VEGFR2 (Franklin et al., 2011). Its binding to VEGFR2 blocks the interactions with its ligands, including VEGF-A, VEGF-C, and VEGF-D, thereby preventing VEGFR2 phosphorylation and downstream consequences such as proliferation, migration, and tube formation of human endothelial cells, finally inhibiting tumor angiogenesis. Ramucirumab was the first approved antibody drug targeting VEGFRs. It is nowadays used in combination with paclitaxel or docetaxel as second-line therapy to treat gastric cancer. The major side effects include hypertension, proteinuria, and thrombocytopenia.

### 2.7 Brolucizumab

Brolucizumab is a newly (in 2019) FDA-approved anti-VEGF agent for the treatment of nAMD (Markham, 2019b). It is a novel humanized single-chain fragment antibody, which inhibits all isoforms of VEGF-A (Motevasseli et al., 2021b). It is composed of only 255 amino acids (molecular mass of approximately 26 kDa), with high solubility and high permeability, which facilitate its delivery from the vitreous into the retina and choroid (Tadayoni et al., 2021). Meanwhile, brolucizumab was reported to be more effective to bind VEGF (binding affinity in a low picomolar range) than other anti-VEGF agents, about 11 times more efficient than aflibercept (Eason et al., 2020; Rahman and Singer, 2020). It is also a long duration anti-nAMD drug, which requires monthly intravitreal administration. Side effects have been reported such as occlusive retinal vasculitis and intraocular inflammation (Witkin et al., 2020). The most common ocular adverse effects are subconjunctival hemorrhage, vitreous floaters, reduced visual acuity, vitreous detachment, and the most common non-ocular side effects are upper respiratory tract infection and urinary tract infection (Motevasseli et al., 2021a).

### 2.8 Faricimab

Faricimab was developed as the first bispecific monoclonal antibody for the treatment of nAMD and DME, which was approved in 2021 (Shirley, 2022). It is the first humanized bispecific antibody designed for treatment of ocular diseases. As a bispecific heterodimeric antibody, faricimab has different light chains in each of the Fab regions, one binds to VEGF and the other one binds to angiopoietin-2 (Ang-2) (Nair et al., 2022). Ang-2 is another pro-angiogenic factor, which activates Tie2 receptor and its downstream signaling to promote cell survival and vascular stability (Joussen et al., 2021). Tie2 receptor has an important role in inflammation and vascular destabilization (Heier et al., 2021). Faricimab can neutralize circulating VEGF and Ang-2 simultaneously to prevent VEGF-induced angiogenesis and restore vascular stability by decreasing leakage, inflammation, and neovascularization (Heier et al., 2022; Liberski et al., 2022). In the faricimab molecule, the Fc fragment, which is the crystallizable region of antibodies binding to cell receptors and complement proteins, has also been modified to reduce undesirable immune system response (Nair et al., 2022). Phase 2 trials in patients with nAMD demonstrated that the safety and efficacy of faricimab was comparable with ranibizumab. Interestingly, the IVT injection interval of faricimab can be up to 16-week, thus reducing the burden for patients. However, it has been argued that the high dose of faricimab injected (6 mg) might be a major reason for the decrease in number of IVT injections, and that the extent to which Ang-2 blockade contributed to the therapeutic efficacy is still unclear (Cao et al., 2023).

All the above molecules are biomolecules targeting the extracellular VEGF-VEGFR interaction. At the beginning, they were targeting only VEGF-A and later they were developed to neutralize more VEGF family members. Two of them are used to treat cancers, in combination with cytotoxic chemical drugs (Table 1). Reported adverse effects are hypertension, fatigue, diarrhea, and complications due to weaker wound-healing ability. Six of them are used to treat retinopathies (Table 1). The intraocular injection (IVT) of these drugs leads to additional adverse effects in the eye including eye pain, corneal edema, blurred vision/reduced vision acuity. Prior to these drugs, many of retinal diseases patients were treated with ablative laser therapy. Therefore, the anti-VEGF therapy has improved vision outcomes and quality of life for millions of people in a revolutionary manner. In behalf of the high specificity of antibodies, antibody-drug conjugates (ADC) are actually in great boom of investigation, but no ADC has been approved for antiangiogenic therapy yet.

## 3 Inhibitors targeting VEGFR tyrosine kinase (TK)

Protein kinases are enzymes that catalyze the transfer of the phosphate group from ATP to hydroxylated amino acids, such as serine, threonine, and tyrosine (Al-Obeidi et al., 1998; Cohen, 2002). They play significant roles in the pathogenesis of autoimmune, inflammatory, nervous and cardiovascular diseases, notably in malignancies. Consequently, they are nowadays one of the most important drug targets (Roskoski, 2023). A protein kinase domain is a protein region with conserved structure containing the catalytic function of protein kinases (Arter et al., 2022). VEGFR1, VEGFR2, and VEGFR3 are structurally related TK receptors, responsible for physiological and pathological vascularization. They have two intracellular TK domains responsible for the activation of VEGFRs and the initial signal transportation of VEGFR downstream signaling. Thus, the VEGFR TK has become one of the most important targets for the development of anti-angiogenic drugs. So far, a total of 72 drugs have been approved by the FDA targeting different protein kinases, most of them are multikinase inhibitors (Roskoski, 2023). Among them, 13 drugs directly inhibit the activity of VEGFR tyrosine kinases (Table 2), along with other receptor RTKs including FGFRs, PDGFRs, TGF-βRs, etc., All these small molecules are approved for anticancer but not for retinopathies treatment.

**TABLE 2:** Approved anti-angiogenic drugs targeting VEGFR tyrosine kinase.

Name	Chemical structure	Targets	First approval	Current indications
Sorafenib	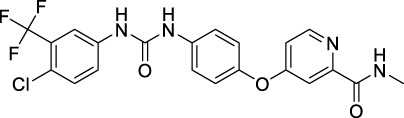	VEGFR1/2/3 and c-Kit/FLT3/RET/PTC/PDGFR-β	2005 by FDA	RCC, HCC, DTC, Thyroid cancer
Sunitinib	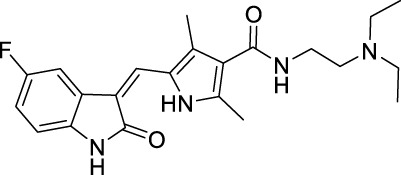	VEGFR1/2/3 and PDGFRα/β, c-Kit, CSF1R, RET, FLT3	2006 by FDA	RCC, GIST, Pancreas neuroendocrine tumor
Pazopanib	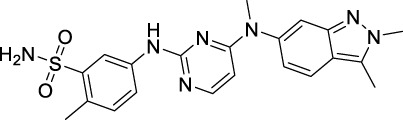	VEGFR1/2/3 and PDGFRα/β, FGFR-1/3, c-Kit	2009 by FDA	RCC, STS
Vandetanib	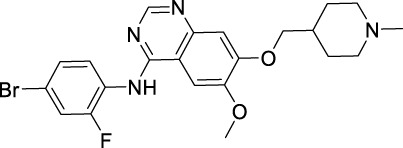	VEGFR2/3 and EGFR, RET, BRK, TIE2, EPH	2011 by FDA	MTC
Axitinib	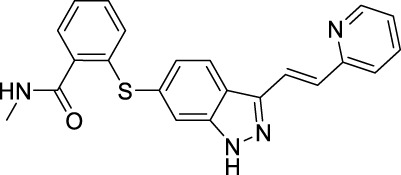	VEGFR1/2/3	2012 by FDA	RCC
Cabozantinib	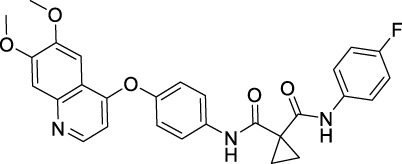	VEGFR1/2/3 and Tie2, c-Met, c-Kit, RET, AXL	2013 by FDA	MTC, RCC, HCC
Regorafenib	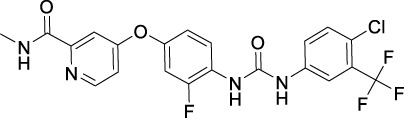	VEGFR1/2/3 and PDGFRα, FGFR1/2, BRAF	2012 by FDA	CRC, GIST, HCC
Apatinib	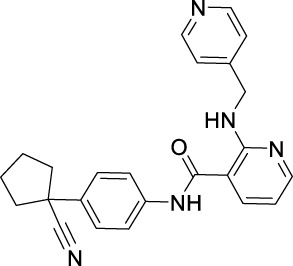	VEGFR2 and c-Src, c-Kit	2014 by NMPA	Gastric cancer
Nintedanib	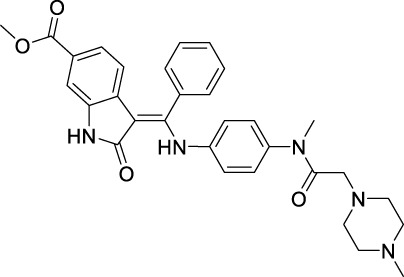	VEGFR1/2/3 and FGFR1/2, PDGFRα/β	2014 by FDA	NSCLC
Lenvatinib	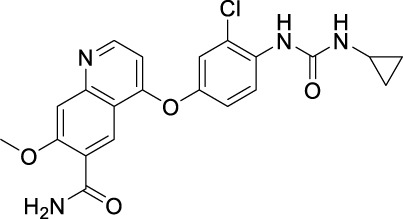	VEGFR1/2/3 and FGFR1/2/3/4, RET, c-Kit, PDGFRα	2015 by FDA	DTC, Thyroid cancer, RCC, HCC, Endometrial carcinoma
Anlotinib	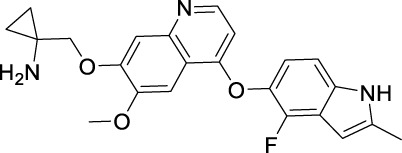	VEGFR2/3 and PDGFRα/β, RET, FGFRs, c-Kit	2018 by NMPA	NSCLC, STS, SCLC
Fruquintinib	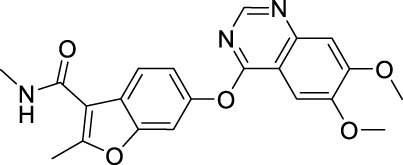	VEGFR1/2/3	2018 by NMPA	CRC
Tivozanib	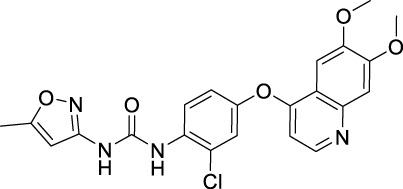	VEGFR1/2/3	2017 by EMA, 2021 by FDA	RCC

### 3.1 Sorafenib

Sorafenib is the first approved, in 2004, anti-angiogenic inhibitor targeting all three VEGFRs (VEGFR1-3) as well as other RTKs, including the stem cell-factor receptor (c-Kit), FLT3, the glial cell-line derived neurotrophic factor receptor (GDNF), PDGFR-β, and the papillary thyroid carcinomas (PTC) (Wilhelm et al., 2006; Keating, 2017). It contains a structure of unsymmetrical substituted urea and was initially approved by the FDA for the treatment of advanced RCC in 2005, and subsequently approved for the treatment of advanced HCC in 2007, for differentiated thyroid carcinoma (DTC) in 2013 and for thyroid cancer in 2014 (Escudier et al., 2019). Sorafenib exhibits a dual mechanism of action by inhibiting VEGFRs and PDGFR signaling and thus inhibiting the RAF/MEK/extracellular signal-regulated kinase (ERK) pathway to reduce tumor angiogenesis and to induce tumor cell apoptosis (Ramakrishnan et al., 2010).

### 3.2 Sunitinib

Sunitinib is the second approved anti-angiogenic TKI, approved 1 year after sorafenib, in 2006, for the treatment of RCC and imatinib-resistant gastrointestinal stromal tumor (GIST) (Motzer et al., 2017). It is an oral oxindole derivative, multi-targeting RTKs, such as VEGFR1/2/3, PDGFRα/β, c-Kit, colony stimulating factor receptor Type 1 (CSF1R), RET (rearranged during transfection), and FLT3 (Hao and Sadek, 2016; Schmid and Gore, 2016). Sunitinib also showed potent antitumor activity against neuroendocrine tumors and obtained its further approval in 2011 for the treatment of progressive, well-differentiated pancreatic neuroendocrine tumors (pNET) in adult patients with unresectable locally advanced or metastatic disease (Blumenthal et al., 2012).

### 3.3 Pazopanib

Pazopanib is a second-generation multitargeted TK inhibitor against VEGFR1/2/3, PDGFRα/β, FGFR-1/3, and c-Kit (Sonpavde and Hutson, 2007; Sloan and Scheinfeld, 2008). It is an indazolylpyrimidine that competes with adenosine triphosphate for binding to the TK domains of these receptors and prevents the ATP-induced receptor activation. It inhibits purified VEGFR1/2/3 kinases with IC_50_ of 10, 30, and 47 nM (Miyamoto et al., 2018). Pazopanib was developed as a therapeutic agent against various types of cancers, however, it has been so far approved only for the treatment of RCC and advanced soft-tissue sarcoma (STS).

### 3.4 Vandetanib

Vandetanib is an anilinoquinazoline derivative antineoplastic kinase inhibitor, which inhibits the activities of VEGFR2/3, EGFR, RET, BRK, TIE2, and EPH (Morabito et al., 2010). It showed a lesser extent inhibition to VEGFR1 TK. In 2011, vandetanib was approved by the FDA for the treatment metastatic medullary thyroid carcinoma (MTC) of adult patients, which makes it as the first effective systemic therapy for MTC (Tsang et al., 2016). This indication was attributed to its inhibitory effect on RET, which is a TK hyperactivated by mutations in MTC (Yoh et al., 2017). Vandetanib can cause some common adverse effects including nausea, diarrhea, hypertension, headache and some significant cardiac toxicities, which restrains its application in other cancers (Ton et al., 2013).

### 3.5 Axitinib

Axitinib is also a second-generation TK inhibitor that works by selectively inhibiting VEGFRs (VEGFR1/2/3), thus blocking angiogenesis, tumor growth and metastases (Kessler et al., 2012). It was reported to be more potent than sunitinib and sorafenib in inhibiting the TK activities of VEGFRs (Goldstein et al., 2010; Gross-Goupil et al., 2013). It is a diarylthioether derivative, which received FDA-approval in 2012 for RCC, particularly as second-line treatment (Bellesoeur et al., 2017).

### 3.6 Cabozantinib

Cabozantinib is a diarylether derivative multitargeted TK inhibitor, targeting VEGFRs, Tie2, c-Met, c-Kit, RET, AXL, etc., (Ruiz-Morales and Heng, 2016; Osanto and van der Hulle, 2018). It is a non-specific TK inhibitor with potent kinase inhibitory activity. Its high inhibitory effect on RET promoted the initial approval of cabozantinib for the treatment of MTC in 2012 (Nagilla et al., 2012). Subsequently, it was approved by the FDA for the treatment of RCC in 2016, and for the treatment of HCC in 2019 (Al-Salama and Keating, 2016; El-Khoueiry et al., 2021).

### 3.7 Regorafenib

Regorafenib is a fluoro-derivative of sorafenib developed by Bayer. It is also an oral multikinase inhibitor, as sorafenib, but with additional kinase targets, such as PDGFRα, FGFR1/2 and BRAF (Wilhelm et al., 2011). Regorafenib received first FDA approval in 2012 (Crona et al., 2013). The FDA expanded its indication to advanced GIST 1 year later and to HCC in 2017 (Grothey et al., 2020).

### 3.8 Apatinib

Apatinib, with substituted 2-amino nicotinamide core, was developed by a Chinese pharmaceutical company, Hengrui Medicine, and approved by the NMPA of China for the treatment of advanced gastric cancer in 2014 (Roviello et al., 2016). It was granted the second approval by NMPA for the second-line treatment of advanced HCC in 2021. It was reported as an anti-angiogenic inhibitor targeting VEGFR2, c-Src, and c-Kit (Scott, 2018).

### 3.9 Nintedanib

Nintedanib is an indolinone derivative multikinase inhibitor targeting VEGFR1/2/3, FGFR1/2, and PDGFRα/β. Thus, drug was developed from a program of searching angiogenesis inhibitors targeting VEGFR2 at Boehringer Ingelheim (Roth et al., 2015). Nintedanib showed an IC_50_ of 34 nM, 21 nM and 13 nM for VEGFR1, VEGFR2, and VEGFR3 TK in an adenosine 5′-triphosphate (ATP) assay (Hilberg et al., 2008). As a potent kinase inhibitor, nintedanib has been evaluated in several solid tumors, including NSCLC, ovarian cancer, CRC, RCC, and HCC (Roth et al., 2015). However, it gained its first approval from the FDA only for the treatment of idiopathic pulmonary fibrosis in 2014. And later, it gained approval as a second-line combination therapy with docetaxel for patients with NSCLC (Alshangiti et al., 2018).

### 3.10 Lenvatinib

Lenvatinib is a 4-O-aryl quinoline derivative acting as a multiple TKs inhibitor targeting VEGFR1/2/3, FGFR1/2/3/4, c-Kit, RET, and PDGFRα (Inoue et al., 2012; Cabanillas and Habra, 2016). It was firstly approved in 2015 for the treatment of DTC and thyroid cancer. A randomized, open-label, phase III trial in patients with unresectable HCC showed that patients treated with lenvatinib had a similar overall survival (OS) as sorafenib treated patients, but with significant improvements in objective response rate, progression-free survival and time to progression (Ikeda et al., 2016; Al-Salama et al., 2019). These results promoted the approval of lenvatinib as the first-line treatment for HCC in 2018 (Baxter et al., 2018).

### 3.11 Anlotinib

Anlotinib, a quinolen-indole derivative, was co-developed by Jiangsu Chia-Tai Tianqing Pharmaceutical and Advenchen Laboratories in China (Syed, 2018). It is a multikinase inhibitor targeting VEGFR2/3, PDGFRα/β, FGFRs, c-Kit, and RET. It showed significant inhibitory effects on angiogenesis and tumor growth (Gao et al., 2020). Anlotinib has demonstrated potent efficacy and a sufficient safety in many malignant cancers in clinical studies (Han et al., 2018a; Han et al., 2018b). It received its first approval from NMPA of China in May 2018, as a third-line treatment for refractory advanced NSCLC. Subsequently, it was approved to treat advanced STS and to treat relapsed small cell lung cancer (SCLC) in 2019 and 2020, in China.

### 3.12 Fruquintinib

Fruquintinib, a quinazoline-benzofuran derivative, is a highly selective kinase inhibitor of VEGFRs with potent anti-angiogenic activities (Burki, 2018). It was developed to treat solid tumors involved with pathological angiogenesis (Shirley, 2018). Fruquintinib gained the first global approval from NMPA of China in September 2018, for the treatment of CRC.

### 3.13 Tivozanib

Tivozanib is a quinoline-urea derivative that specifically targets kinase domains of VEGFR1, VEGFR2, and VEGFR3 (Jacob et al., 2020). It was firstly approved in the European Union by EMA as a first-line treatment for advanced RCC in adult patients (Kim, 2017). Tivozanib was later approved by the FDA for the treatment of relapsed or refractory RCC in March 2021.

Since the first report of small molecules inhibiting EGFR TK activity (Yaish et al., 1988), the development of inhibitors of RTK (receptor tyrosine kinase) has been a hot topic, leading to the first approved TK inhibitor (TKI) imatinib in 2001. The first drug targeting VEGFR, sorafenib, was approved some years later in 2005. A dozen of TKIs targeting angiogenic receptors, mainly VEGFRs, are now on the market in anticancer therapy. They are beneficial for certain types of cancers with significant improvement of patient’s outcomes. However, as all other small chemical drugs, they have also undesirable side effects, such as abdominal pain, nausea, diarrhea, fatigue, hand-foot skin reaction, etc. The most severe side effects are cardiovascular and renal adverse effects (Goldman et al., 2021; Van Wynsberghe et al., 2021).

## 4 Inhibitors targeting VEGFR downstream signaling

VEGFR downstream signaling is a complex process, activated by cross phosphorylation of the VEGFR TK domains. The angiogenesis signals are transported by different signaling pathways, resulting in cell survival, proliferation, migration and tube formation. The RAS/RAF/MEK/ERK pathway and the PI3K/AKT/mTOR pathway are the most well studied VEGFR downstream signaling pathways (Simons et al., 2016). Other signaling pathways, such as NCK/p38/MAPKAPK2/3 pathway and SRC/FAK/Paxillin pathway are also reported to be involved in VEGFR downstream signaling (McMullen et al., 2005; Sun et al., 2012). These signaling proteins are also kinase proteins, which are called non-receptor kinase proteins. The signaling transport is a linear cascade of kinase protein interactions and phosphorylation. As a result, inhibitor targeting these non-receptor protein kinases can also inhibit VEGFR induced angiogenesis. Here, we summarize 16 approved drugs targeting VEGFR downstream RAS/RAF/MEK/ERK signaling pathway and PI3K/AKT/mTOR signaling pathway. However, these drugs are not commonly classified as anti-angiogenic inhibitors, because their protein kinase targets are also involved in many other cellular processes. Inhibiting non-receptor kinase proteins of RAS/RAF/MEK/ERK signaling pathway and PI3K/AKT/mTOR signaling pathway can induce not only antiangiogenesis effect, but also other biological effects, such as antiproliferation, cytotoxicity, apoptosis, etc. Currently, these drugs are mainly used in anticancer therapy (Table 3).

**TABLE 3:** Approved drugs targeting VEGFR downstream signaling, classified by their targets.

Name	Chemical structure	Targets	First approval	Current indications
Sotorasib	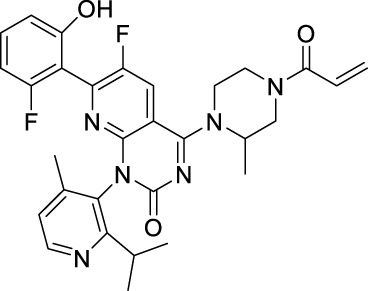	KRAS	2021 by FDA	NSCLC
Adagrasib	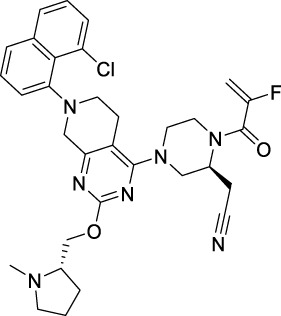	KRAS	2022 by FDA	NSCLC
Vemurafenib	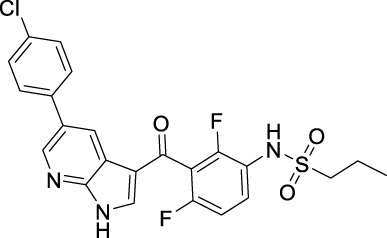	BRAF	2011 by FDA	Melanoma
Dabrafenib	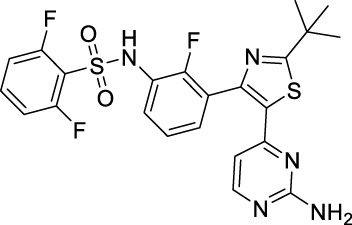	BRAF, CRAF	2013 by FDA	Melanoma, NSCLC, ATC
Encorafenib	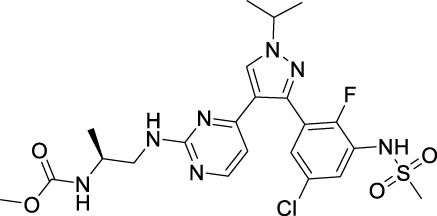	BRAF	2018 by FDA	Melanoma
Trametinib	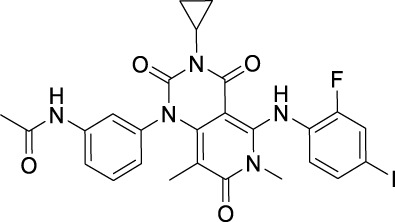	MEK1/2	2013 by FDA	Melanoma, NSCLC, ATC
Cobimetinib	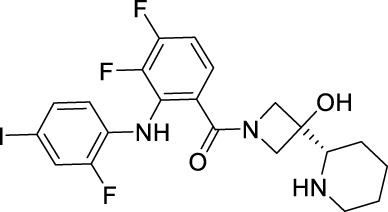	MEK1/2	2015 by FDA	Melanoma
Binimetinib	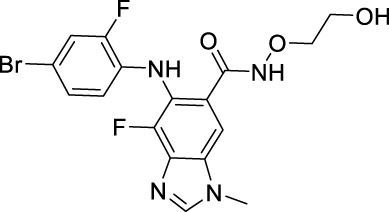	MEK1/2	2018 by FDA	Melanoma
Selumetinib	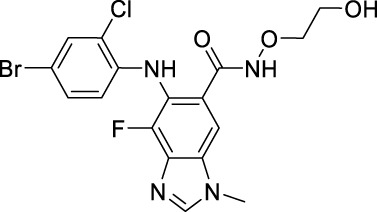	MEK1/2	2020 by FDA	Neurofibromatosis type 1, Plexiform neurofibromas
Idelalisib	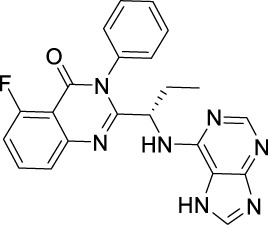	PI3Kδ	2014 by FDA	CLL, Follicular lymphoma
Copanlisib	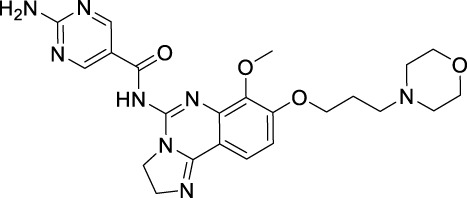	Pan-PI3K	2017 by FDA	Follicular lymphoma
Duvelisib	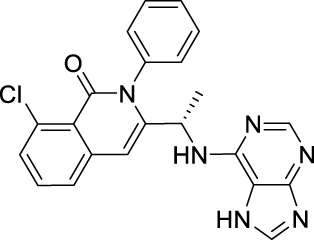	PI3Kγ/δ	2018 by FDA	CLL, SLL, Follicular lymphoma
Alpelisib	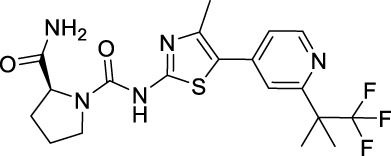	PI3Kα	2019 by FDA	Breast cancer
Temsirolimus	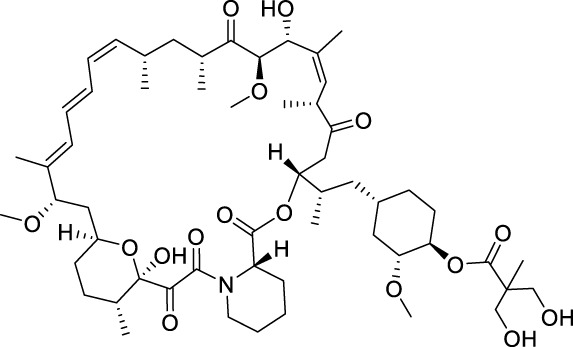	mTOR	2007 by FDA	RCC
Everolimus	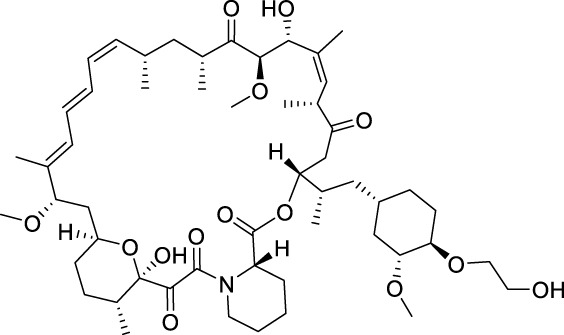	mTOR	2009 by FDA	RCC, pancreatic cancer, breast cancer
Sirolimus	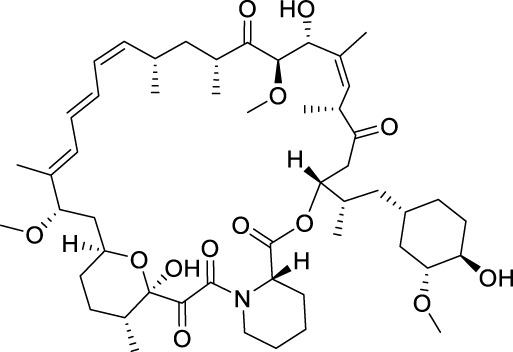	mTOR	2015 by FDA	LAM

### 4.1 Inhibitors targeting RAS/RAF/MEK/ERK signaling pathway

The Rat sarcoma virus (RAS)/rapidly accelerated fibrosarcoma (RAF)/mitogen-activated extracellular signal-regulated kinase (MEK)/extracellular signal regulated kinase (ERK)/signaling pathway is a well-known signaling pathway that regulates cell survival, growth, and proliferation in normal cells and cancer cells. Among them, RAS is a small GTPase that binds tightly to GTP with picomolar affinities. The mutants KRAS and NRAS are frequently found in cancers (Liu et al., 2023). RAF is a serine/threonine kinase directly activated by RAS. It includes the mutants ARAF, BRAF, and CRAF, also frequently found in cancers (Yoshino et al., 2019). MEK1/2 is a dual-specificity protein kinase activated by RAF, which can subsequently phosphorylate ERK1/2 and transport signaling into nucleus to promote cell survival and proliferation (Degirmenci et al., 2020).

RAS was thought to be an undruggable target because of its picomolar binding affinity to GTP, until the discovery of irreversible inhibitors targeting KRAS G12C (Zheng et al., 2022). Recently, two small molecules KRAS inhibitors, sotorasib and adagrasib were granted FDA approval in 2021 and in 2022 for the treatment of KRAS G12C mutant non-small cell lung cancer (Rathod et al., 2023).

For RAF, especially BRAF mutations have been more commonly identified in melanomas and other malignancies. Three BRAF inhibitors, vemurafenib (Tsai et al., 2008), dabrafenib (Hauschild et al., 2012), and encorafenib (Li et al., 2016) have been approved for the treatment of non-resectable BRAF V600E or V600K mutant melanoma and anaplastic thyroid cancer (Liu et al., 2019). Sorafenib (Table 2), which is a multi RTK inhibitor, also showed inhibitory activity to BRAF (Casadei Gardini et al., 2016).

With the success of BRAF inhibitors, MEK and ERK were subsequently investigated as potential targets of RAS/RAF/MEK/ERK signaling pathway. Four MEK inhibitors, trametinib, cobimetinib, binimetinib and selumetinib have been approved by FDA for the treatment of unresectable or metastatic melanoma alone or in combination with BRAF inhibitors. Trametinib is a reversible, highly selective, non-ATP competitive allosteric inhibitor of MEK1/2 by binding to unphosphorylated MEK1 and MEK2 with high affinity (Salama and Kim, 2013) and blocks the catalytic activity of MEK1/2. It was first approved by the FDA in 2013 as monotherapy for the treatment of melanoma (Wright and McCormack, 2013). Currently, it is more often used as combination therapy with BRAF inhibitors (Table 3) for the treatment of unresectable or metastatic melanoma harboring BRAF V600E and/or V600K mutation (Roskoski, 2018). Cobimetinib and binimetinib were granted FDA approval also as combination therapy with BRAF inhibitors for the treatment of patients with unresectable or metastatic melanoma with BRAF V600E/V600K mutation (Eroglu et al., 2016). Selumetinib, having a similar structure as binimetinib (Table 3), has been approved in 2020 by the FDA as monotherapy for the treatment of Neurofibromatosis type 1 (NF-1) (Markham and Keam, 2020). It is a more selective and potent second-generation allosteric MEK1/2 inhibitor (Hedayat et al., 2022). Selumetinib is still under investigation for a variety of solid tumors, for example, phase II trials for endometrial cancer and non-small cell lung cancer (Coleman et al., 2015; Seto et al., 2018), and a phase III trial in differentiated thyroid (Ho et al., 2022), and melanoma (Carvajal et al., 2018). Currently, no ERK inhibitor has yet been reported to be used in the clinic.

Inhibitors targeting the RAS/RAF/MEK/ERK signaling pathway have made brilliant progress in the clinic, promoting the development of a variety of novel inhibitors targeting this cascade. New KRAS G12C inhibitors such as GDC-6036, JDQ443, LY3537982, MK-1084, JAB-21822, BI-1823911, and D-1553 are in pre-clinical and clinical development (Lorthiois et al., 2022; Parikh et al., 2022; Xu et al., 2022; Li et al., 2023). Novel RAF inhibitors have been developed as well, such as PF-07284890 (ARRY-461), a small molecule inhibitor targeting BRAF V600E and V600K(Bouhana et al., 2021); XL281 (BMS-908662), a potent and selective inhibitor of wild-type and mutant RAF kinases (Dickson et al., 2015); and RO5126766, which was evaluated as a dual RAF/MEK allosteric inhibitor with a novel coumarin skeleton based structure (Martinez-Garcia et al., 2012; Wada et al., 2014). Recently, two novel MEK inhibitors, AZD-8330 and GDC-0994, are under clinical trials for the treatment of advanced solid malignancies (Cohen et al., 2013; Weekes et al., 2020).

### 4.2 Inhibitors targeting PI3K/AKT/mTOR signaling pathway

The phosphatidylinositol 3-kinase (PI3K)/V-AKT murine thymoma viral oncogene homolog (AKT)/mammalian target of rapamycin (mTOR) signaling cascade is also a well-studied signaling pathway that controls normal cells and cancer cells growth, proliferation, and survival (Huang et al., 2022). The PI3Ks family contains three classes of lipid kinases, PI3Ks class I, PI3Ks class II and PI3Ks class III, according to the subunits and substrates structures. Among them, PI3K class I is the major isoform implicated in cancer, which can be further divided into class IA (contains PI3Kα, PI3Kβ, and PI3Kδ) and class IB (contains PI3Kγ). PI3Ks can be activated by RTKs (including VEGFRs) and GPCRs. PI3Ks catalyze the phosphorylation of phosphatidylinositol and promote the transfer from PIP2 to PIP3, which subsequently activates AKT with two phosphorylation sites (Chalhoub and Baker, 2009). Phosphorylated AKT adjacently induces mTOR activation, which results in cell growth, cell survival, inhibition of apoptosis, increased glucose metabolism, protein synthesis, and further signal transduction (Li et al., 2022). Over activation of the PI3K/AKT/mTOR signaling increases not only tumors progress, but also the drug resistance of tumors (Guerrero-Zotano et al., 2016). As an important VEGFR downstream signaling pathway, PI3K/AKT/mTOR signaling pathway becomes an attractive target for developing antiangiogenesis and antitumor targeted drugs.

The study of PI3K inhibitors mainly focuses on inhibitors targeting the four isoforms of the class I PI3Ks (α, β, γ, and δ). Idelalisib was the first selective PI3Kδ inhibitor approved by the FDA in 2014 for the treatment of relapsed or refractory chronic lymphocytic leukemia (CLL) (Shah and Mangaonkar, 2015). It showed acceptable safety and durable antitumor activity accompanied with improved quality-of-life outcomes in clinical trails. Copanlisib is a pan-PI3K inhibitor more potent than idelalisib, approved in 2017 for the treatment of adult patients with relapsed follicular lymphoma and a treatment history of at least two prior systemic therapies. Compared to idelalisib, copanlisib adopts a flat conformation better-fitting in the adenine-binding pocket, and further extends into a deeper pocket of the catalytic p110 subunit (Krause et al., 2018). It has IC_50_ values in the single digit nanomolar range against class I PI3K-α, β, γ, and δ isoforms (Liu et al., 2013). Duvelisib is a dual inhibitor of PI3Kγ and PI3Kδ (Vangapandu et al., 2017). It also binds to the ATP-binding pocket of p110 (Vangapandu et al., 2017). The FDA has approved duvelisib for the treatment of relapsed or refractory CLL or small lymphocytic lymphoma (SLL) in adult patients who had at least two prior therapies (Blair, 2018). Alpelisib is a selective inhibitor targeting class I PI3Kα with high *in vitro* affinity (Furet et al., 2013; James et al., 2015). This drug is indicated as combination therapy with fulvestrant, an estrogen receptor antagonist, for the treatment of hormone receptor (HR)-positive, human epidermal growth factor receptor-2 (HER2)-negative breast cancer in patients with a PI3KCA mutation (Markham, 2019a). More PI3K inhibitors are undergoing preclinical and clinical evaluation, such as ZSTK474 analogues, pilaralisib and IPI-549. They showed a favorable safety profile and antitumor activity in different cancers (Matulonis et al., 2015; Wang et al., 2016; Liu et al., 2020).

The development of AKT inhibitors seems more challenging. No AKT inhibitor has been yet approved as antiangiogenesis or antitumor agent. Most of them are still undergoing preclinical and clinical evaluation. Among these molecules, the ATP-competitive AKT inhibitors, such as ipatasertib, capivasertib, afuresertib, and uprosertib have shown potent antitumor activity in clinical trials (McKenna et al., 2018; Alzahrani, 2019). For example, ipatasertib in combination with abiraterone and prednisone/prednisolone showed efficacy in patients with HR-positive and HER2-negative locally advanced unresectable or metastatic breast cancer in a phase III trial (Sweeney et al., 2021); A phase II trial showed that the combination of capivasertib and fulvestrant significantly prolonged progression-free survival of patients with metastatic breast cancer (Jones et al., 2020); Afuresertib showed favorable safety, pharmacokinetics, and clinical activity as monotherapy in multiple myeloma in a phase I trial (Spencer et al., 2014); Uprosertib showed satisfying safety and good tolerability in patients with solid tumors (Aghajanian et al., 2018). However, these AKT inhibitors still need more clinical evidence before being approved in clinical use.

mTOR (mammalian target of rapamycin) inhibitors are the first compounds developed to target the PI3K/AKT/mTOR signaling pathway. Rapamycin analogs are the first-generation mTOR inhibitors, which inhibit only mTORC1 (mTOR complex 1) but not mTORC2 (Benjamin et al., 2011). Three rapamycin macrolide analogs with slight molecular modifications, temsirolimus, everolimus, and sirolimus have been approved by the FDA in 2007, in 2009 and in 2015, respectively. They are used in the treatment of various cancers, including RCC, breast cancer, pancreatic cancer and lymphangioleiomyomatosis (LAM). The second-generation mTOR inhibitors, such as sapanisertib, vistusertib, and GDC-0349, binding competitively to the ATP-binding pocket of mTOR with high binding affinity, are undergoing preclinical and clinical trials (Benjamin et al., 2011; Hsieh et al., 2012; Pei et al., 2013). Several dual PI3K/mTOR inhibitors have also been discovered and developed, such as bimiralisib, dactolisib and gedatolisib (Shi et al., 2018; Collins et al., 2021; Shor et al., 2022). They showed simultaneous ATP-binding domain inhibition to PI3K and mTOR, which induces promising PI3K/AKT/mTOR signaling cascade blockage. Nonetheless, none of them has been approved in clinical use yet.

## 5 Conclusion and perspectives

Angiogenesis plays an important role in several diseases’ progression, such as malignant tumors and retinopathies. The VEGF/VEGFR axis, including VEGF/VEGFR interaction, VEGFR tyrosine kinase phosphorylation and VEGFR downstream signaling, is the key process of angiogenesis. Indeed, anti-angiogenic therapy has become one of the most effective clinical therapeutic approaches for DME, nAMD and multiple cancers. Dozens of inhibitors targeting the VEGF/VEGFR axis have been approved and used in clinic. Many more are undergoing preclinical and clinical trials. In this review, we classify these drugs as inhibitors of the VEGF/VEGFR interaction, inhibitors of VEGFR TK and inhibitors of VEGFR downstream signaling.

Inhibitors of VEGF/VEGFR interaction are mainly biological molecules, such as antibodies, antibody Fab and fusion proteins. They are highly specific agents acting on the VEGF/VEGFR interaction blockade by effectively binding to VEGFs or VEGFRs. Among them, the anti-VEGF agents, such as brolucizumab, aflibercept, ranibizumab, conbercept, and faricimab, are currently the unique drugs approved to treat retinopathies including DME and nAMD. Anti-VEGF agents can also be used as antitumor drugs, such as bevacizumab. However, ramucirumab, which binds specifically to the extracellular domain of VEGFR2 and blocks the interaction of VEGFR2 with its ligands (VEGF-A, VEGF-C, and VEGF-D) was approved only for cancer treatment, but not in retinopathies therapy. It seems that neutralizing specifically VEGFs induces milder antiangiogenic effects than blocking VEGFRs. Blocking VEGFRs or inhibiting VEGFR TK domain or inhibiting VEGFR downstream signaling inhibits not only cell growth, proliferation, migration, but also induces apoptosis, which is accompanied with cytotoxicity. Recently, we have been working on development of peptide inhibitors to block the VEGF/VEGFR interaction (Wang et al., 2017; Wang et al., 2019; Ye et al., 2023). Some of our peptides showed effective activity to block the VEGF/VEGFR interaction, and induced inhibition of HUVEC proliferation, migration, and formation of microtubes. They showed antitumor effects on a xenografted mouse model (Wang et al., 2021).

Inhibitors of VEGFR TK are mostly multiple kinase inhibitors, due to the similarity of kinase catalytic domains of RTKs, such as VEGFRs, FGFRs, Kits, PDGFRs and TGFRs. They inhibit not only VEGFR induced angiogenesis but also other RTKs induced cell growth, proliferation, survival, migration, and tube formation. They are mainly used as antitumor therapy.

The VEGFR downstream signaling is complex. The key proteins and enzymes involved can be activated not only by phosphorylation of VEGFR TK, but also by other RTKs. They induce cell cycle regulation, invasion and metastasis. We summarized here the clinical used drugs targeting RAS/RAF/MEK/ERK signaling pathway and PI3K/AKT/mTOR signaling pathway. Most of them showed anti-proliferative activity and cytotoxicity by blocking the essential proteins such as KRAS, BRAF, MEK1/2, PI3Kα, β, γ, δ, and mTOR. Inhibitors targeting these signaling pathways will cut off all the signaling transportation in the cascade, certainly suppressing the cell survival, growth, proliferation and migration. However, it is also accompanied with side effects, such as cytotoxicity and drug resistance.

All anti-angiogenic agents suffered a major challenge, the drug resistance. The biologic drugs targeting VEGF/VEGFR interaction and small molecules targeting VEGFR TK and downstream signaling all come across drug resistance after a period of monotherapy in treatment of cancer or retinopathies. As a result, combination therapy is often applied, especially in cancer treatment to achieve desired results and to minimize drug resistance. For example, clinical trials showed that bevacizumab combined with erlotinib significantly prolonged PFS compared with monotherapy in treatment of patients with EGFR-positive advanced NSCLC (Deng et al., 2022). Moreover, some signaling pathway inhibitors are only approved in combination therapy, such as binimetinib, cobimetinib, encorafenib and alpelisib (Markham, 2019a; Logenthiran et al., 2020). Improved combination compositions are undergoing clinical trials including multi-targets, dual-functions, etc., (Li et al., 2022).

In conclusion, the inhibitors targeting VEGF/VEGFR axis are essential in regulating the pathological angiogenesis in cancers and eye diseases. They will continue to be an intense drug research topic because of their clear mechanism and effective outcomes in the clinic.

## Figures and Tables

**Table:** 

**ATC**	anaplastic thyroid cancer
**Ang-2**	angiopoietin-2
**BC**	breast cancer
**CLL**	chronic lymphocytic leukemia
**CRC**	colorectal cancer
**DR**	diabetic retinopathy
**DTC**	differentiated thyroid carcinoma
**DME**	diabetic macular edema
**ECs**	endothelial cells
**EGF**	epidermal growth factor
**FGF**	fibroblast growth factor
**GEJ**	gastro-esophageal junction
**GIST**	gastrointestinal stromal tumor
**GBM**	glioblastoma multiforme
**HCC**	hepatocellular carcinoma
**IVT**	intravitreal
**LAM**	lymphangioleiomyomatosis
**MTC**	medullary thyroid carcinoma
**nAMD**	neovascular age-related macular degeneration
**NRP1 and NRP2**	neuropilins 1 and 2
**NSCLC**	non-small-cell lung cancer
**OC**	ovarian cancer
**PlGF**	placental growth factor
**PDGF**	platelet-derived growth factor
**PTC**	papillary thyroid carcinomas
**RTKs**	receptor tyrosine kinases
**RCC**	renal cell carcinoma
**RET**	rearranged during transfection
**RVO**	retinal vein occlusion
**ROP**	retinopathy of prematurity
**SCLC**	small cell lung cancer
**STS**	soft-tissue sarcoma
**TGF**	transforming growth factor
**TK**	tyrosine kinase
**VEGF**	vascular endothelial growth factor
